# Composite diagnostic criteria are problematic for linking potentially distinct populations: the case of frailty

**DOI:** 10.1038/s41598-020-58782-1

**Published:** 2020-02-13

**Authors:** Yi-Sheng Chao, Chao-Jung Wu, Hsing-Chien Wu, Hui-Ting Hsu, Lien-Cheng Tsao, Yen-Po Cheng, Yi-Chun Lai, Wei-Chih Chen

**Affiliations:** 1 Independent researcher, Québec, Canada; 2 Département d’informatique, Université du Québec à Montréal, Québec, Canada; 3 Department of Internal Medicine, Taipei Hospital, Ministry of Health and Welfare, New Taipei City, Taiwan; 4 Department of Surgery, Changhua Christian Hospital, Changhua County, Taiwan; 5 Division of Chest Medicine, Department of Internal Medicine, National Yang-Ming, University Hospital, Yi-Lan, Taiwan; 6 Department of Chest Medicine, Taipei Veterans General Hospital, Faculty of Medicine and Institute of Emergency and Critical Care Medicine, School of Medicine, National Yang-Ming University, Taipei, Taiwan

**Keywords:** Geriatrics, Epidemiology

## Abstract

Composite diagnostic criteria are common in frailty research. We worry distinct populations may be linked to each other due to complicated criteria. We aim to investigate whether distinct populations might be considered similar based on frailty diagnostic criteria. The Functional Domains Model for frailty diagnosis included four domains: physical, nutritive, cognitive and sensory functioning. Health and Retirement Study participants with two or more deficiencies in the domains were diagnosed frail. The survival distributions were analyzed using discrete-time survival analysis. The distributions of the demographic characteristics and survival across the groups diagnosed with frailty were significantly different (p < 0.05). A deficiency in cognitive functioning was associated with the worst survival pattern compared with a deficiency in the other domains (adjusted p < 0.05). The associations of the domains with mortality were cumulative without interactions. Cognitive functioning had the largest effect size for mortality prediction (Odds ratios, OR = 2.37), larger than that of frailty status (OR = 1.92). The frailty diagnostic criteria may take distinct populations as equal and potentially assign irrelevant interventions to individuals without corresponding conditions. We think it necessary to review the adequacy of composite diagnostic criteria in frailty diagnosis.

## Introduction

Medical diagnoses are classifications and the objective of diagnosis is to accurately and efficiently identify patients’ health problems^1^. To form diagnoses, diagnostic criteria are often applied and consist of certain items or measures used to determine diseases or syndromes^1–3^. In some cases, a single diagnostic criterion is sufficient to identify the target condition, such as hypernatremia that is determined based on the blood sodium levels^4^. In other circumstances, composite diagnostic criteria involving multiple items or measures are necessary for the diagnosis of diseases or conditions^2,3,5–8^. The reasons for the use of composite diagnostic criteria include disease complexity^6–8^, extensive involvement of multiple biomarkers in the disease development^9^, intermittent presence of the symptoms or signs^10^, and unknown pathological base of the target conditions or diseases^2^. In general, the use of composite diagnostic criteria often results from the fact that single measures or items may not be sufficient to describe or determine cases. For example, currently there is no validated single-item measure to determine the degree of frailty^11,12^. Three of the most commonly used frailty syndromes requires four to 70 items in the composite criteria to diagnose frailty statuses^12,13^.

However, composite diagnostic criteria, including the three frailty diagnoses commonly used by researchers or clinicians^12^, may be subject to several limitations. First, bias or measurement error can be generated while rating each criterion or summing the final scores for the composite diagnostic criteria^12^. There is evidence to show that bias can occur when continuous measures are converted to categorized diagnostic criteria^12,14^ or when the total numbers of the criteria are top censored^12^. As a result, 73.7% of the variances of the frailty index diagnosed according to the Biological Syndrome Model can be explained by bias alone^12^. Second, the diagnostic criteria may not represent the original theory or evidence base. For example, the accumulation of deficits has been used as one type of the composite diagnostic criteria for frailty^12^. Once the number of deficits reaches certain thresholds, individuals can be diagnosed with frailty^13^. However, the number of deficits according to the Burden Model is in fact highly associated with cardiovascular disease^12^, while the theory that motivates the diagnosis of frailty does not emphasize cardiovascular disease^12^. The composite diagnostic criteria may fail to reflect the theory or evidence base. Lastly, the development of composite diagnostic criteria may not be associated with pathological findings. For example, lung function has not been considered in the biology of the development of frailty^15^. However, the deficits related to lungs can be used for frailty diagnosis^12,16^.

Besides these recently identified limitations to the composite diagnostic criteria of frailty^12^, we are concerned that composite diagnostic criteria may falsely identify a group of patients with a common health problem to assign adequate treatment. In other words, distinct populations with different health problems may be arbitrarily linked together based on composite diagnostic criteria. For example, we are not sure whether the patients diagnosed with frailty based on the deficiencies in nutritive and physical functioning are similar to those diagnosed based on cognitive deficiencies and sensory problems^17^. If these two groups are similar, it is ethical and clinically obligatory to treat them in the same way. If not, we think it may be unethical and harmful to treat these two groups as equal. For this concern, this study aims to understand the differences in individual characteristics and survival patterns between population groups that are all diagnosed with the same condition, frailty. If possible, we are also interested in how the frailty domains are associated with survival patterns across the groups.

## Results

**Figure 1:**
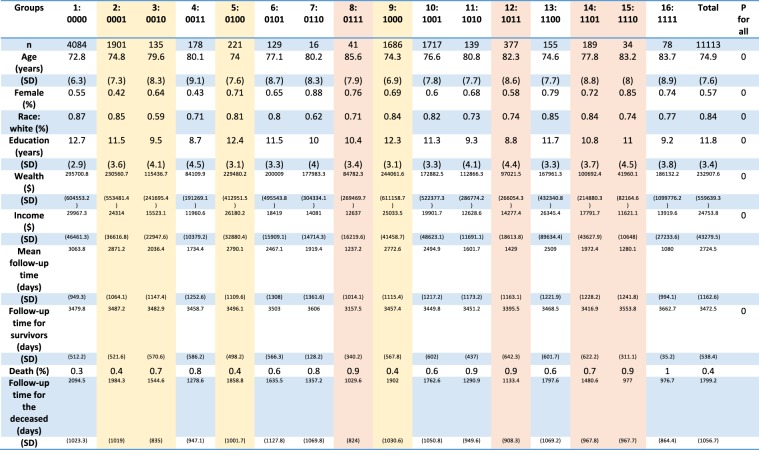
The characteristics of the 16 population groups categorized based on the four frailty domains of the Functional Domains Model. Note: the 16 groups are based on the combinations of presence or absence of deficiencies in the four frailty domains. The groups are labelled with serial numbers from one to 16 and code names that identify the deficiencies in the frailty domains. The four domains of the Functional Domains Model for the diagnosis of frailty are (1) physical functioning, (2) nutritive functioning, (3) cognitive functioning, and (4) sensory problems. Individuals with two or more deficiencies in the four domains are considered frail. For example, the code, 0011, represents the absence of deficiencies in the physical functioning and nutritive functioning domains with the presence of deficiencies in the cognitive functioning and sensory problems domains. The groups with one deficiencies in the four domains are in yellow shade and those with three deficiencies are in red shade.

The characteristics of the 16 groups categorized based on the combinations of the four frailty domains in 2004 were compared in Fig. 1. The distributions of age in years, sex, race, education in years, per capita income and per capita wealth were significantly different across 16 groups (p < 0.05 for all). When tested among those with two or more deficiencies in any four frailty domains (diagnosed with frailty), the distributions of age, education in years, per capita wealth, and per capita income were not significantly different (p > 0.05 for all), while sex and race distributions were significantly different (p < 0.05 for both).

**Figure 2:**
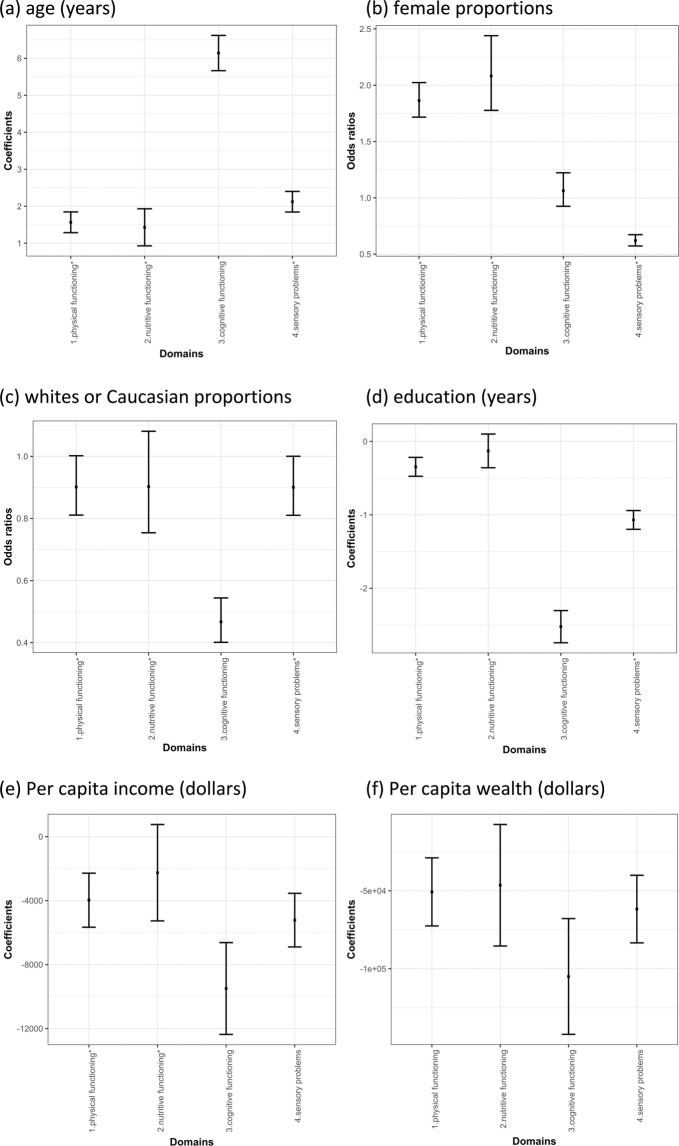
Regression coefficients of the four frailty domains for estimating individual characteristics. (**a**) coefficients for age in years. (**b**) odds ratios for female proportions. (**c**) odds ratios for the proportions of white or Caucasian. (**d**) coefficients for education in years. (**e**) coefficients for per capita income. (**f**) coefficients for per capita wealth. Note: those without any deficiencies in the four frailty domains were the baseline group for comparison. The coefficients were estimated via linear regression and the four frailty domains were the independent variables. The odds ratios were estimated via logistic regression with the four frailty domains as independent variables. The ranges are 95% confidence intervals. *significantly different from the association with Domain 3) cognitive functioning.

The associations between the frailty domains and the characteristics were quantified using regression models. In Fig. 2a, the four frailty domains were significantly associated with older age, 1.4 years (95% CI = 0.9 to 1.9) for Domain 2) nutritive functioning to 6.1 years (95% CI = 5.7 to 6.6) for Domain 3) cognitive functioning, while the age estimate was 72.8 years (95% CI = 72.6 to 73.0) for those without any deficiency. In Fig. 2b, the OR of Domain 3) cognitive functioning was not significantly associated with female proportions, compared to those without any deficiencies. Domain 1) physical functioning and Domain 2) nutritive functioning were associated with higher proportions females, OR = 1.8 (95% CI = 1.7 to 2.0) and 2.1 (95% CI = 1.8 to 2.4) respectively. Domain 4) sensory problems was associated with lower proportions of females, OR = 0.6 (95% CI = 0.57 to 0.67). In Fig. 2c, three domains were not significantly associated with the higher or lower proportions of whites or Caucasian compared to those without any deficiency in the four domains: Domain 1) physical functioning, Domain 2) nutritive functioning, and Domain 4) sensory problems. Domain 3) cognitive functioning was associated with lower proportions of whites or Caucasian, OR = 0.47 (95% CI = 0.40 to 0.54). In Fig. 2d, Domain 2) nutritive functioning was not significantly associated with differences in years of education compared to those without any deficiency in the four frailty domains. The other three domains were associated with less years of education from 0.3 years less (95% CI = −0.5 to −0.2) for Domain 1) physical functioning to 2.5 years less (95% CI = −2.7 to −2.3) for 3) cognitive functioning. In Fig. 2e, Domain 2) nutritive functioning was not significantly associated with differences in per capita income compared to those without deficiency in the four domains. The other three domains were associated with less per capita income, from $3971.9 less (95% CI = −5,661.3 to −2,282.5) for Domain 1) physical functioning to $9,496.6 less (95% CI = −12,368.3 to −6,624.9) for Domain 3) cognitive functioning. For per capita wealth in Fig. 2f, the four domains were associated with lower per capita wealth than those without any deficiency in the four domains, from $46,423.1 less (95% CI = −85,419.3 to −7,429.0) for Domain 2) nutritive functioning to $104,958.9 less (95% CI = −142,105.8 to −67,811.9) for Domain 3) cognitive functioning.

Based on the findings in Fig. 2, populations with a deficiency in Domain 3) cognitive functioning were significantly different in most characteristics. Compared to the magnitudes of the association with Domain 3) cognitive functioning, the associations of the other three frailty domains with age, female distributions, white or Caucasian distributions, and education in years were significantly different (adjusted p < 0.05). The magnitudes of the associations with per capita income were not different between Domain 3) cognitive functioning and Domain 4) sensory problems (adjusted p > 0.05), while the differences in the magnitudes of the associations remained significant compared to Domain 1) physical functioning and Domain 2) nutritive functioning (adjusted p < 0.05 for both). The magnitude of the association between per capita wealth and Domain 3) cognitive functioning was not significant different from those between per capita wealth and the other three domains (adjusted p > 0.05 for all).

### Correlation between the frailty domains

**Figure 3:**
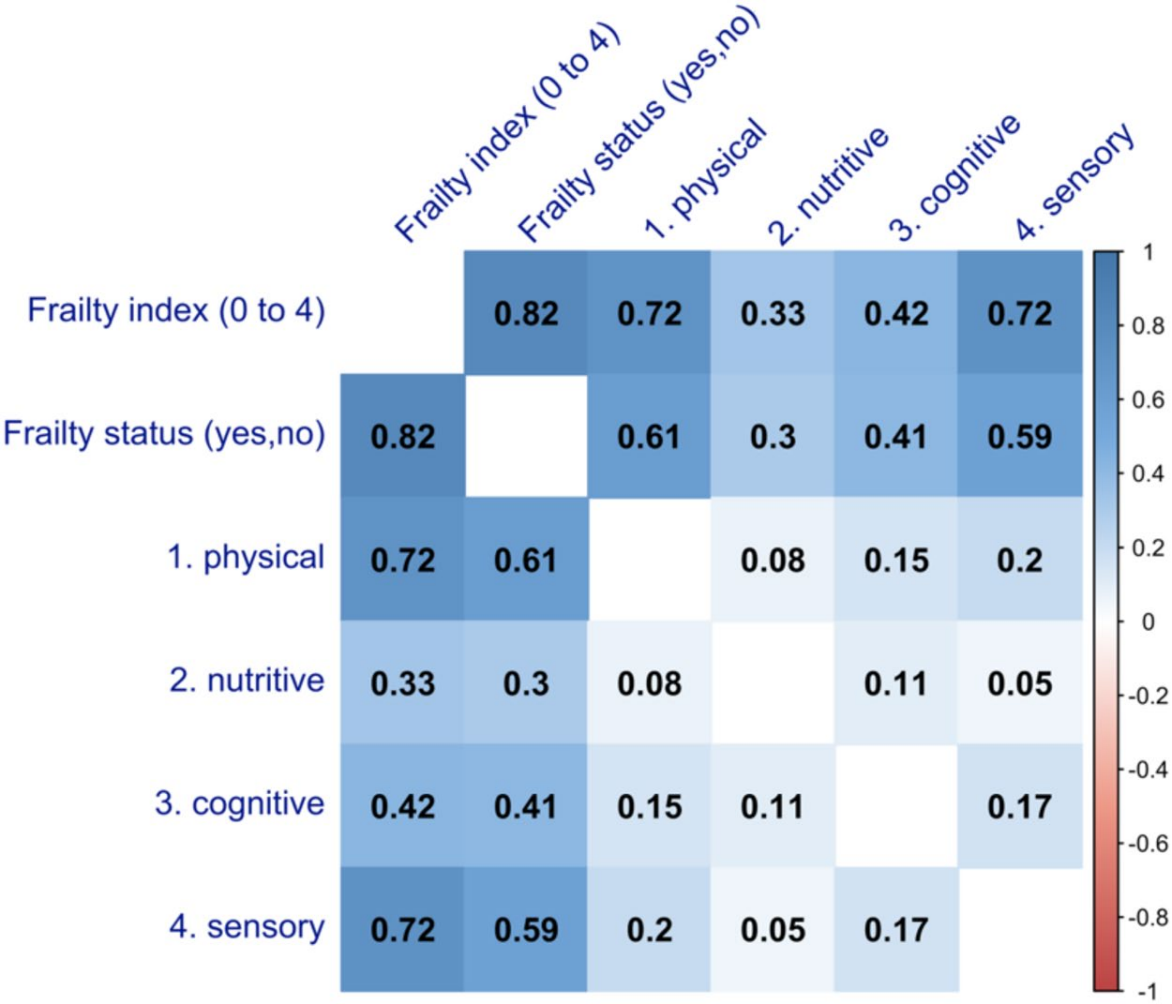
The correlations between the four frailty domains in the Functional Domains Model. Numbers in the cells = Spearman’s correlation coefficients, ranging from 1 to −1; (1) physical = physical functioning domain in the Functional Domains Model; (2) nutritive = nutritive functioning domain; (3) cognitive = cognitive functioning domain; (4) sensory = sensory problems domain.

When the four domains were assessed for the correlation between each other, the Spearman’s rank correlation coefficients were shown in Fig. 3. The correlation coefficients indicated very weak or weak correlation and ranged from 0.05 [between Domain 2) nutritive functioning and Domain 4) sensory problems] to 0.20 [between Domain 1) physical functioning and Domain 4) Sensory problems]. Frailty status and frailty index were highly correlated, coefficient = 0.82. Frailty index seemed more correlated with the four frailty domains than frailty status.

### Survival patterns

**Figure 4:**
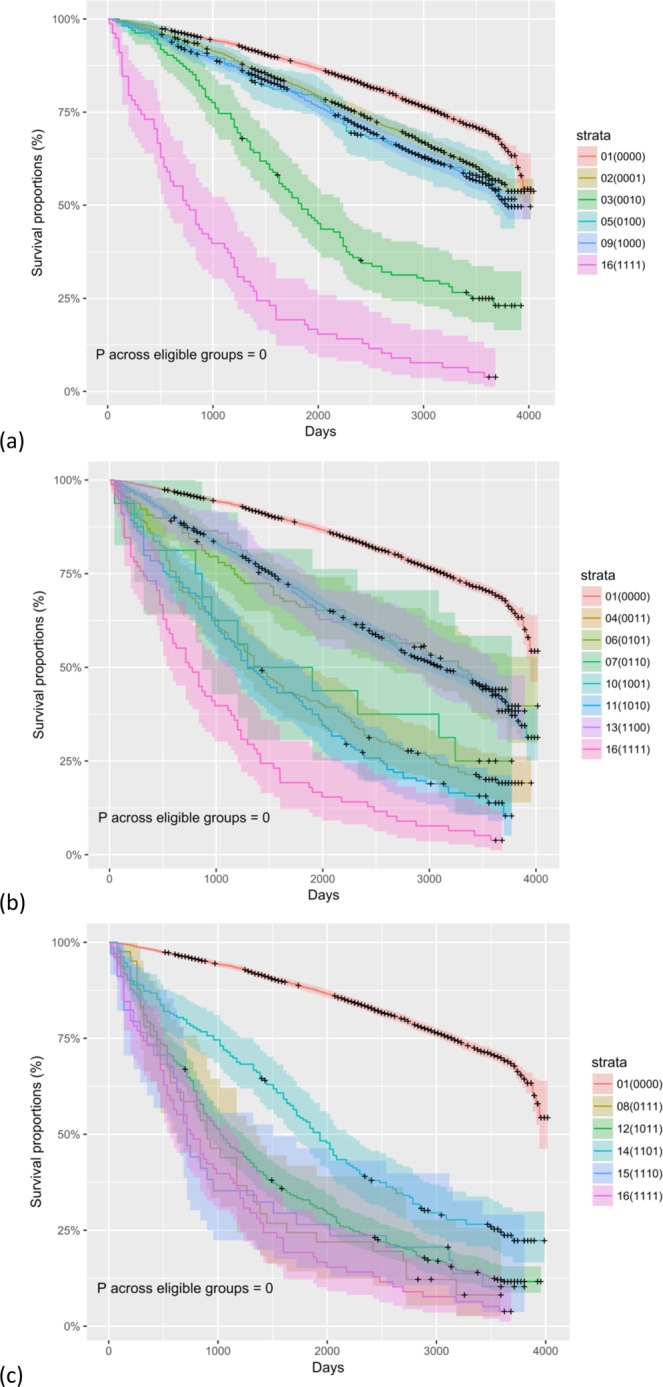
The survival curves of the 16 groups categorized based on the combinations of the four frailty domains in the Functional Domains Model. (**a**) Groups diagnosed with one deficiency in the four frailty domains. (**b**) Groups diagnosed with two deficiencies in the four frailty domains. (**c**) Groups diagnosed with three deficiencies in the four frailty domains. Note: p values derived from log-rank tests to examine the null hypothesis that there was no difference in the survival distributions of the eligible groups. The eligible groups are those labelled in the subtitles and two groups, those not diagnosed with any frailty domains (0000) and those diagnosed with all four frailty domains (1111), are not eligible for respective tests. Population groups are labelled with serial numbers and the codes that represent the combinations of the four frailty domains in the Functional Domains Model. Zero represents the absence of the frailty domains and one the presence. The frailty domains are in the following sequences: physical functioning, nutritive functioning, cognitive functioning, and sensory problems. For example, the code, 0011, represents the absence of domain (1) physical functioning and domain (2) nutritive functioning and the presence of domain (3) cognitive functioning and domain (4) sensory problems.

The survival curves of all 16 groups were plotted together separately in Fig. 4. The survival curves were significantly different across 16 groups, groups with any one deficiency in the four domains, groups with any two, groups with any three, and groups with two or more (p < 0.05 for all, see Appendix 1 for details).

### Groups with one deficiency in the four frailty domains

Unexpectedly, the survival patterns of the four groups with one deficiency in the four frailty domains were significantly different. The group that had no deficiency in any frailty domains had the largest proportion of individuals surviving throughout the follow-up in Fig. 1 and their survival curve was above those of the other 15 groups in Fig. 4. The curve of the group with four deficiencies in the frailty domains had the lowest proportion of individuals surviving and their survival curve was below the other 15. The survival curves of the four groups with a deficiency in the frailty domains were illustrated along with those of the groups with none or four deficiencies in the four domains in Fig. 4a. The survival distributions of the four groups were significantly different (p < 0.05). The survival curve of the group with a deficiency in Domain 3) cognitive functioning was the lowest of the four.

**Figure 5:**
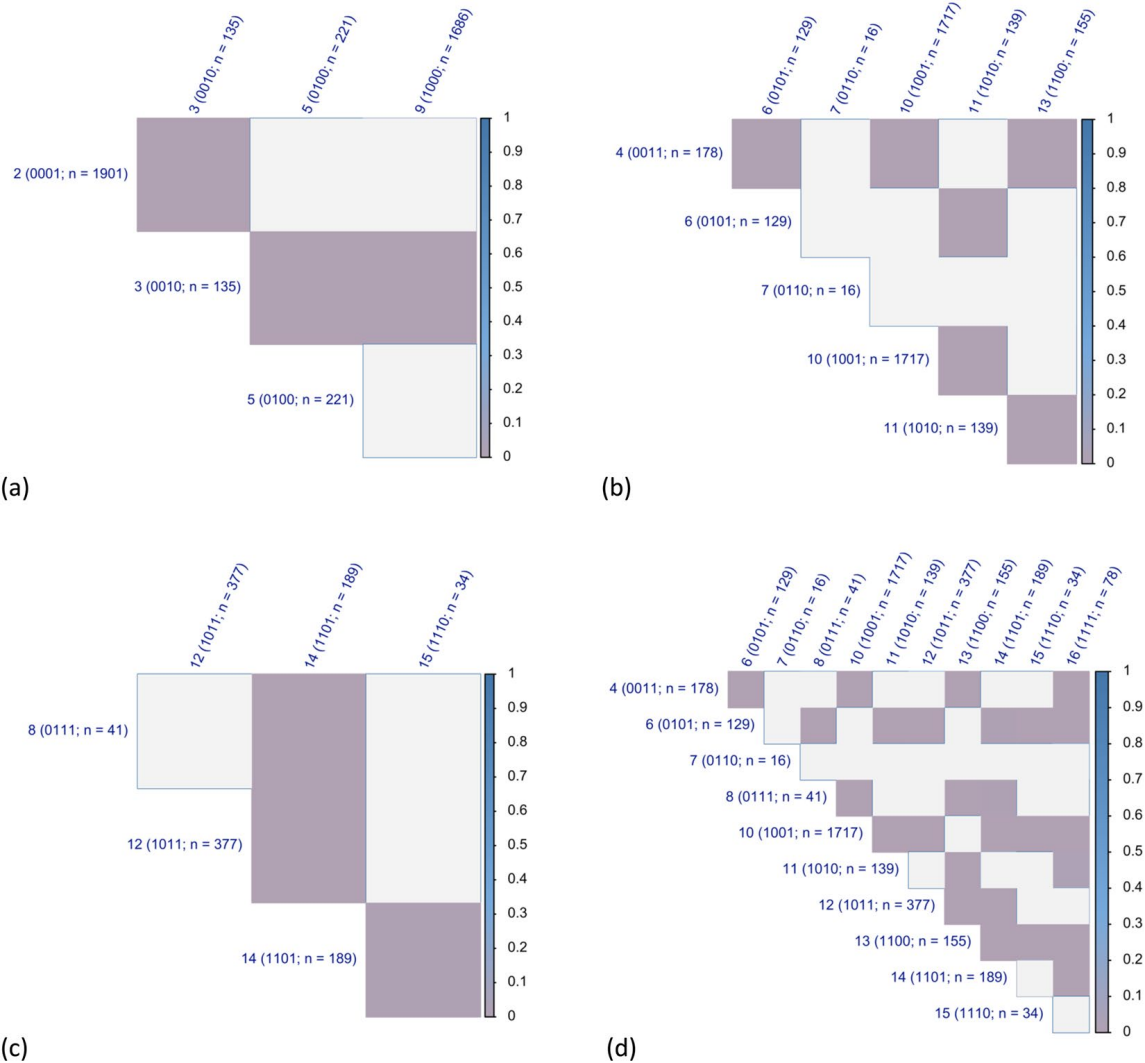
The p values of the comparisons in the survival distributions of the population groups categorized according to the 16 combinations of the four frailty domains in the Functional Domains Model. (**a**) Groups diagnosed with one deficiency in the four frailty domains. (**b**) Groups diagnosed with two deficiencies in the four frailty domains. (**c**) Groups diagnosed with three deficiencies in the four frailty domains. (**d**) Groups diagnosed with two or more deficiencies in the four frailty domains. Note: grey cells = adjusted p < 0.05. The p values derived from log-rank tests to examine the null hypothesis that there is no difference in the survival distributions of the eligible groups. The p values were adjusted for multiple comparisons. The blank cells represent insignificant survival differences. Population groups are labelled with serial numbers and the codes that represent the combinations of the four frailty domains in the Functional Domains Model. Zero represents the absence of the frailty domains and one the presence. The frailty domains are in the following sequences: physical functioning, nutritive functioning, cognitive functioning, and sensory problems. For example, the code, 0011, represents the absence of physical functioning and nutritive functioning domains with the presence of cognitive functioning and sensory problems domains.

**Figure 6:**
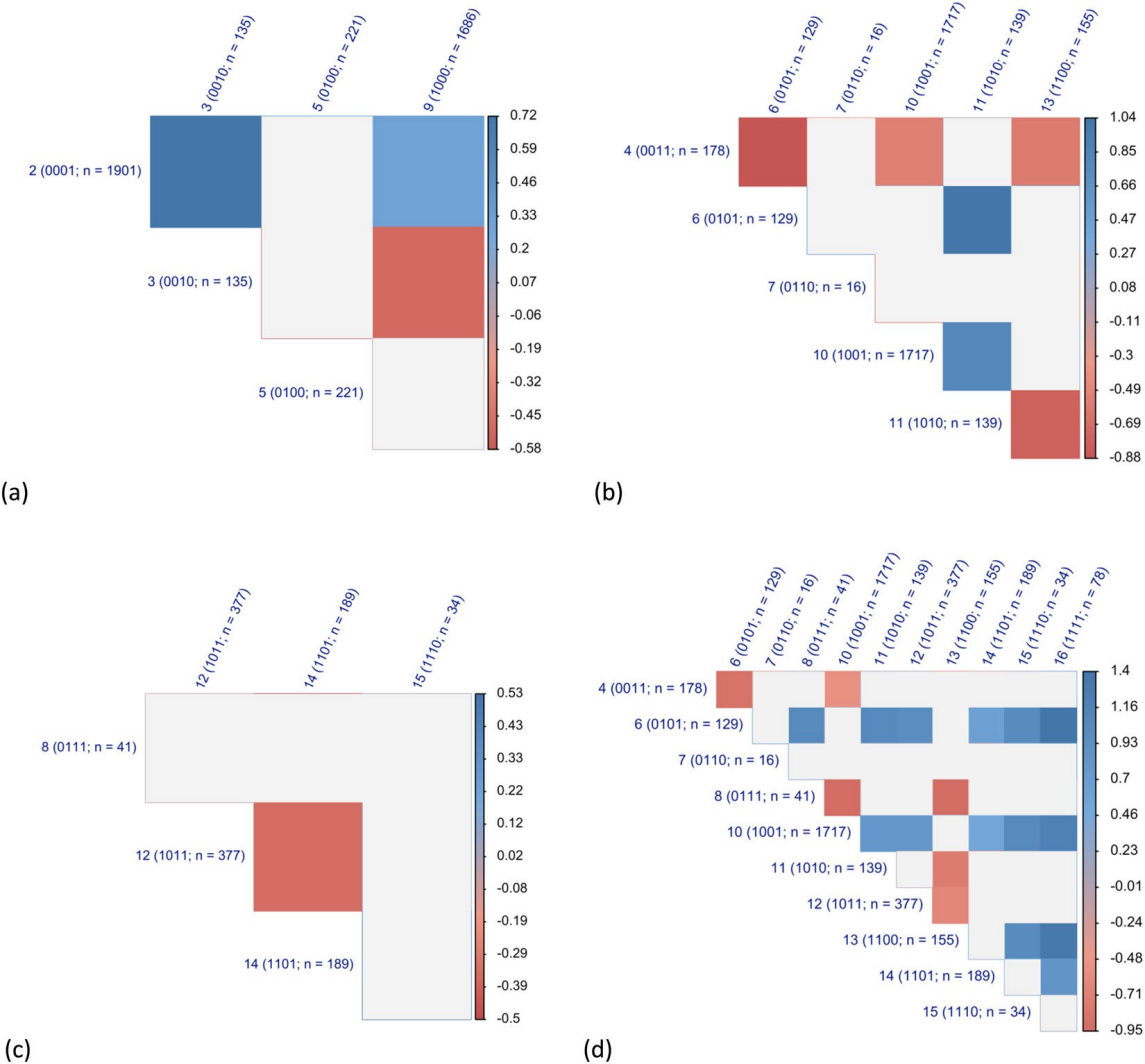
The regression coefficients of the comparisons in the survival distributions of the population groups categorized according to the 16 combinations of the four frailty domains in the Functional Domains Model. (**a**) Groups diagnosed with one deficiency in the four frailty domains. (**b**) Groups diagnosed with two deficiencies in the four frailty domains. (**c**) Groups diagnosed with three deficiencies in the four frailty domains. (**d**) Groups diagnosed with two or more deficiencies in the four frailty domains. Note: regression coefficients derived from discrete-time survival analysis to examine the null hypothesis that there is no difference in the survival distributions of the eligible groups, while demographic characteristics are adjusted, including age, sex, race, years of education, per capita income and per capita wealth. The blank cells represent insignificant survival differences. The groups to the left of the graph were the baseline. If the coefficients are negative, the group to the left of the graph has higher mortality risk. Population groups are labelled with serial numbers and the codes that represent the combinations of the four frailty domains in the Functional Domains Model. Zero represents the absence of the frailty domains and one the presence. The frailty domains are in the following sequences: physical functioning, nutritive functioning, cognitive functioning, and sensory problems. For example, the code, 0011, represents the absence of physical functioning and nutritive functioning domains with the presence of cognitive functioning and sensory problems domains.

The survival curve of individuals with a deficiency in Domain 3) cognitive functioning was significantly different from those of the other groups with one deficiency. In fact, when the four survival curves were compared to each other, the survival distribution of the group with a deficiency in Domain 3) cognitive functioning was significantly different from the other three in Fig. 5a (p < 0.05 for eligible comparisons, adjusted for multiple comparisons). However, when demographic characteristics were taken into account in survival analysis, we found that the group with a deficiency in Domain 2) nutritive functioning had the survival distribution not different from the other three groups in Fig. 6a (adjusted p > 0.05 for eligible comparisons).

### Groups with two deficiencies in the four frailty domains

Unexpectedly, the groups with two deficiencies in the four frailty domains were significantly different in the survival patterns. In Fig. 4b, the survival curves of the six groups with two deficiencies in the four frailty domains were within the area of the two curves of those with none or four deficiencies. The survival distributions of the six groups were significantly different (p < 0.05, see Appendix 1 for details). The curves of the three groups that did not have a deficiency in Domain 3) cognitive functioning overlapped: Group 6 with deficiencies in Domain 2) nutritive functioning and Domain 4) sensory problems, Group 10 with deficiencies in Domain 1) physical functioning and Domain 4) sensory problems, and Group 13 with deficiencies in Domain 1) physical functioning and Domain 2) nutritive functioning. The other three groups that had deficiency in Domain 3) cognitive functioning had curves lower than the other three: Group 4 with deficiencies in Domain 3) cognitive functioning and Domain 4) sensory problems, Group 7 with deficiencies in Domain 2) nutritive functioning and Domain 3) cognitive functioning, and Group 11 with deficiencies in Domain 1) physical functioning and Domain 3) cognitive functioning. However, when the survival distributions were compared between groups, Group 7 consisting of only 16 HRS participants did not have survival distribution significantly different from the other five groups (adjusted p > 0.05 for all eligible comparisons).

The survival curve of individuals with a deficiency in Domain 3) cognitive functioning was significantly different from those of the other groups with two deficiencies. Excluding the comparisons involving Group 7, there were six comparisons between the groups with a deficiency in Domain 3) cognitive functioning and those without, one comparison between the groups with a deficiency in Domain 3) cognitive functioning [Group 4 (code: 1010) and Group 11 (1010)], and three comparisons between the groups without a deficiency in Domain 3) cognitive functioning. In Fig. 5b, only the six comparisons between the groups with or without a deficiency in Domain 3) cognitive functioning were significant (adjusted p < 0.05 for all). In other words, the between-group comparisons showed only the survival distributions between the groups with a deficiency in Domain 3) cognitive functioning and those without were significantly different, while a group of 16 participants did not have a survival distribution significantly different from the other five.

In Fig. 6b, when the demographic characteristics were taken into account in survival analysis, the significant comparisons were the same as those in Fig. 6b. The directions of the regression coefficients showed that the groups with the deficiency in Domain 3) cognitive functioning had higher mortality risk than those without (adjusted p < 0.05 for all).

### Groups with three deficiencies in the four frailty domains

Unexpectedly, the populations with three deficiencies in the four frailty domains were significantly different in the survival patterns. In Fig. 4c, the survival curves of the four groups with three deficiencies in the four frailty domains were within the area of those with none or four deficiencies. The survival distributions of the four groups were significantly different (p < 0.05, see Appendix 1 for details). Group 14 without a deficiency in Domain 3) cognitive functioning had the curve higher than those of the other three groups. In Fig. 5c, the between-group comparisons of the survival distributions involving Group 14 were significant (adjusted p < 0.05 for all). The comparisons between the groups with a deficiency in Domain 3) cognitive functioning were not statistically significant (adjusted p > 0.05 for all). The survival distribution of Group 14 was significantly different from those with a deficiency in Domain 3) cognitive functioning. In Fig. 6c, when demographic characteristics were considered in survival analysis, only the comparison between Group 12 (1011) and Group 14 (1101) was significant (adjusted p < 0.05 for both). Those in Group 12 had higher mortality risk than those in Group 14.

### Groups with two or more deficiencies in the four frailty domains

Unexpectedly, the populations with two or more deficiencies in the four frailty domains were significantly different in the survival patterns. The p values of the between-group comparisons of the groups with two or more deficiencies in the four frailty domains were plotted in Fig. 5d. The survival distribution of Group 7 with only 16 participants was not significantly different from those of the other groups. Except for Group 7, there were five groups with a deficiency in Domain 3) cognitive functioning and four groups without. Among 24 comparisons between the groups with a deficiency in Domain 3) cognitive functioning (Group 4, 7, 8, 11, 12, 15, and 16) and those without (Group 6, 10, 13, and 14), 21 were significant. Among 15 comparisons between the groups with a deficiency in Domain 3) cognitive functioning (Group 4, 7, 8, 11, 12, 15, and 16), two were significant. Among six comparisons between the groups without a deficiency in Domain 3) cognitive functioning, two were significant.

The survival curve of individuals with a deficiency in Domain 3) cognitive functioning was significantly different from those of the other groups without such deficiency. In Fig. 6d, demographic characteristics were considered for the comparisons of the survival distributions. According to the between-group comparisons, the Group 6, 10, and 13 without a deficiency in Domain 3) cognitive functioning and a deficiency in the other three domains had survival advantages compared to the other groups with a deficiency in Domain 3) cognitive functioning. In addition, the comparison between Group 14 (1101) and Group 16 (1111) was significant (adjusted p < 0.05).

### Effect sizes of frailty index, frailty status, frailty domains, and interactions between frailty domains

**Figure 7:**
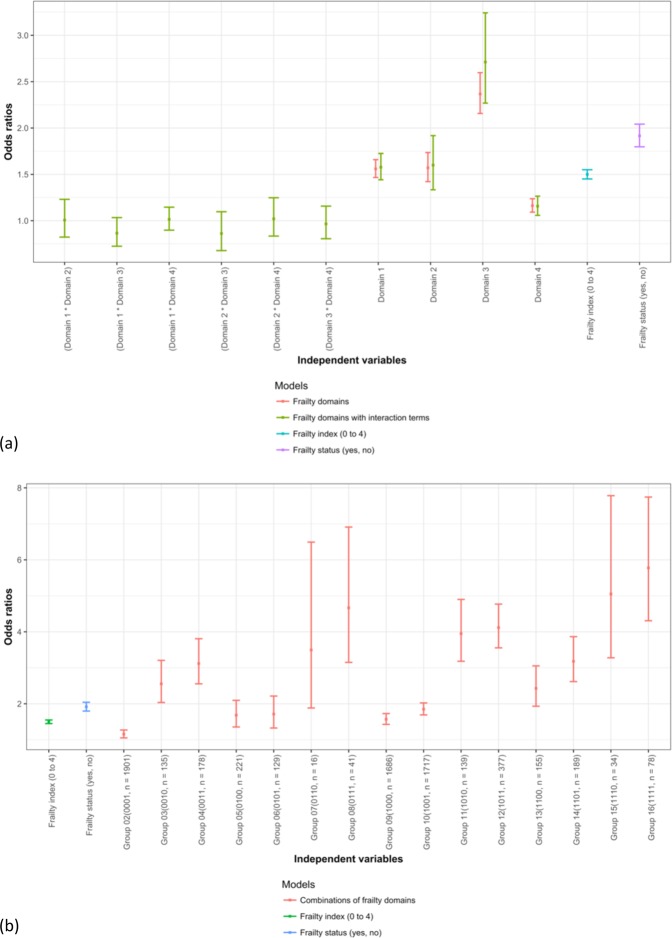
Odds ratios of the frailty index, frailty status, frailty domains, the interaction terms, and 16 frailty groups of frailty domains based on the Functional Domains Model for mortality prediction. (**a**) odds ratios of frailty index, frailty status, frailty domains, and the interaction terms of the frailty domains. (**b**) Odds ratios of frailty index, frailty status, frailty domains, and the interaction terms of the frailty domains. Note: odds ratios greater than one suggesting higher risk of mortality; less than one suggesting lower risk. The ranges represent 95% confidence intervals.

The four domains seemed to predict mortality independently and cumulatively. In Fig. 7, the ORs of the independent variables for mortality prediction were derived while controlling for demographic characteristics in survival analysis. The ORs of frailty index (ranging from zero to four), frailty status (yes if frailty index equal to or greater than two; otherwise not frail), four frailty domains, and the interaction terms of the frailty domains were compared in Fig. 7a, while demographic characteristics were adjusted. The OR of frailty index [1.50, 95% CI = 1.45 to 1.55; see Frailty Index (0 to 4) model in Appendix 3 for details] was close to the average of those of the four frailty domains obtained in a separate regression model (1.56, 1.57, 2.37, and 1.16 for Domain 1, 2, 3, and 4 respectively; log OR of frailty index = −0.91, mean log OR of the Domains = −0.90; see Frailty Domains model in Appendix 3 for details). In Fig. 7a, when interaction terms between the four frailty domains were added, the ORs of the four domains changed slightly and the 95% CIs became wider (see Frailty domains with interaction terms model in Appendix 3 for details). More importantly, the interaction terms were not significant for mortality prediction, while the main effects of the four frailty domains and demographic characteristics were considered in survival analysis (p > 0.05 for all).

The effect size of frailty status for mortality prediction was not larger than all domains. The OR of frailty status, 1.92 [95% CI = 1.80 to 2.04, see Frailty status (yes, no) model in Appendix 3 for details]), was between the ORs of having one or two deficiencies in the frailty index or between those of Domain 2) nutritive functioning and Domain 3) cognitive functioning.

In Fig. 7b, the ORs of the 15 combinations of the presence and absence deficiencies in the four frailty domains were compared with the group without any deficiency (see Combinations of frailty domains model in Appendix 3 for details). The ORs of the groups with one deficiency in the four frailty domains, Group 2, 3, 5, and 9, were similar to those of the four domains, while the 95% CIs were wider. Without significant interactions between the frailty domains for mortality prediction, the ORs of the combinations seemed to be cumulative of the four frailty domains. For example, the log OR of Group 4 (0011), 1.13 (95% CI = 0.94 to 1.34), was similar to the sum of the log ORs of Domain 3) cognitive functioning and Domain 4) sensory problems, 0.86 and 0.15 respectively (95% CIs = 0.77 to 0.95 and 0.09 to 0.21 respectively).

## Discussion

There is evidence to show that populations with distinct characteristics and survival patterns have been considered equally frail and linked together based on the composite diagnostic criteria designed for frailty diagnosis. There are several reasons to this. The frailty domains or the input variables that are often chosen based on theories or data availability are rarely examined for the relationships between them^12^. As a result, frail individuals with a deficiency in cognitive functioning seems to be very different from the other frail individuals without it, in terms of demographic characteristics and survival patterns. The relationships between frailty indices and their input variables are not well studied, either^12^. We found that the nutritive and cognitive functioning was weakly or moderately correlated with the frailty index or status. The frailty index or status seems to represent the physical or sensory functioning better than the other two domains. In fact, in one previous study two of the most commonly used frailty indices are better explained by the biases generated due to data processing than the input variables^12^.

### The problem of linking distinct populations

There are several alarming issues if distinct populations are treated as equal. From a statistical perspective, this is creating an obstacle for precisely estimating the prognosis of the conditions, when these population groups have their own survival trajectories. This has been confirmed by the fact that the frailty domains predict mortality better than frailty status^12^. According to the Functional Domains Model, the effect sizes of four frailty domains have been considered the same and the four domains can be summed to form the frailty index^12^. However, this approach neglects the importance of cognitive functioning and its largest effect size for mortality prediction. In addition, the four frailty domains are independent of each other for mortality prediction, while demographic characteristics are adjusted in survival analysis^12^. The lack of understanding in the independent and cumulative effects of the four frailty domains can lead to statistical models with inferior predictive power.

From a clinical and ethical perspective, irrelevant or even harmful interventions may be prescribed to patients. This is because similar treatment plans will be given to those considered to have a common health problem. In this study, we notice that patients diagnosed with frailty can be individuals without deficiencies in physical functioning or nutritive functioning, because they are diagnosed with frailty for the deficiencies in cognitive functioning and sensory problems. Some “frail” patients without a deficiency in physical functioning can be assigned with various exercise interventions in clinical trials^18^. Other “frail” patients without a deficiency in nutritive functioning may be assigned with a variety of nutritional interventions^19^. The use of composite diagnostic criteria for the diagnosis of frailty can potentially mask the problems that require more attention, especially cognitive deterioration. The treatment plans to frailty are not likely to work for the frailty domains if the fact that these domains are associated with mortality independently and cumulatively are not well perceived^12^.

From a naïve or layperson perspective, it is unclear why having deficiencies in cognitive functioning and sensory problems is the same as having deficiencies in physical and nutritive functioning. This assumption of equal importance is not supported by the effect sizes of the four domains for mortality prediction^12^. We think a patient-oriented frailty measure is required for patients or the public to advance our understanding in frailty and its role in aging^11,12^.

### The problem of composite diagnostic criteria for the diagnosis of frailty

This is the second article to study the problem of composite diagnostic criteria for the diagnosis of frailty. In addition to the issue of linking potentially distinct populations or falsely labelling a “common health problem” to unrelated populations, there are other problems generated by the composite diagnostic criteria.

There are problems identified in the conceptualization, the design and the use of composite diagnostic criteria. The development of composite diagnostic criteria within a specific population can create a diagnosis that is highly sensitive to detect the health problem in this population, but unspecific to other populations^12,20^. The choice of input variables as diagnostic criteria requires rigorous examination of the relationships between the input variables, but theories are the only justification for variable selection in three most commonly used frailty models^12^. It has been found that multiple correlated input variables are summed to create an index defined by the Burden Model that can be simplified with fewer unique variables^12^. In this study, the four frailty domains are not only weakly correlated, but also independent of each other to predict mortality. The benefits of creating a diagnosis of frailty may be outweighed by the drawback derived from summing the numbers of the deficiencies in the four domains: integrating bias to the index, neglecting the importance of cognitive functioning, potentially introducing inappropriate interventions to patients, and ignoring the independence of the four frailty domains for outcome prediction.

For the design of composite diagnostic criteria, two types of data processing are related to the introduction of bias to the final diagnosis, top censoring and categorization of input measures^12,14^. Top censoring of the sum of two or more measures is equivalent to adding noise or bias to the sum of these input variables^12^. Categorization is to distort original data according to certain thresholds^12^. These are one of the fundamental reasons why three commonly used frailty indices can hardly be explained by their input variables chosen by respective theories^12^.

### Research opportunities

Can frailty be seen as a single health problem? We concluded that a good measure was required to define and detect frailty and proposed a new frailty measure that avoids all the issues related to composite diagnostic criteria: a subjective frailty assessment scale^11^. We think it makes better sense to directly ask individuals how they define frailty and how frail they think they are, compared with aggregating a variety of variables into indices that are hardly interpretable.^11^ This frailty scale still requires validation studies in the field and in different populations^11^. In the future, we think an objective frailty assessment tool can be developed based on this.

For frailty measures based on composite diagnostic criteria, we think there may not be enough evidence to support this idea based on the findings and previous research on three of the most commonly used frailty indices^12^. More importantly, can the problems that we demonstrate with frailty indices exist in other composite diagnostic criteria? We think this is very likely for several reasons. First, the basic mechanisms of introducing bias to the diagnosis based on composite diagnostic criteria are prevalent in many other diagnoses. For example, metabolic syndrome has been defined differently and some have proposed establishing the diagnosis based on having at least two of the four conditions^5,6,8^. This step is exactly the same as the diagnosis of frailty out of the four frailty domains according to the Functional Domains Model^12^. The usefulness of metabolic syndrome diagnosis has recently found limited in classifying patients and predicting patient prognosis^5,6,8^. Interestingly, mental disorders, many of which are symptom-based diagnoses, are subject to the weakness that we have identified with three of the commonly used frailty indices. We consider the examination of the diagnoses of mental disorders as the priority. A detailed research proposal has been submitted for review.

Lastly, we think it’s time to think about what’s the value of medical diagnoses. They can be used for statistical^21^ and administrative purposes^22^. They are important for treatment initiation, follow-up, and prognosis estimation^1^. However, there are signs of medicalization or overtreatment that may lead to iatrogenic damage to individuals or prevent them from non-medical interventions^1,23,24^. We are not sure about the magnitudes and exact impact of the problems of composite diagnostic criteria, linking distinct populations and neglecting the most important issues behind the criteria. The consequences need to be assessed with other diseases diagnosed based on composite diagnostic criteria and treated with various interventions.

### Strengths and limitations

There are several strengths to this study. The survival patterns have not been interfered by external interventions. This is because most of the nutritive and exercise frailty interventions were introduced after 2010^18,19,25^. The differences in survival patterns were not subject to most frailty interventions^18,19,25^. The frailty domains or diagnostic criteria are based on a well-implemented longitudinal survey, the HRS. The individuals have been followed for a maximum of 13 years in this data set^12,26–29^. The researchers who were responsible for the survey implementation were not likely to be biased toward or against the diagnosis of frailty. However, there are several limitations to this study. The 16-group categorization leads to few numbers of subjects in certain groups. This is the reason why some of the between-group comparisons are not significant. The applicability of this conclusion needs to be tested with other conditions that are currently diagnosed based on composite diagnostic criteria. For example, many of the mental disorders are diagnosed based on a set of major criteria and the other set of minor criteria^2,3^.

## Conclusions

Frailty can be diagnosed when individuals have two or more deficiencies in the four frailty domains defined by the Functional Domains Model. Eleven of the 16 population groups categorized by the presence and absence of deficiencies in the four frailty domains were diagnosed with frailty. The 11 groups are significantly different in survival patterns and most demographic characteristics. The groups without a deficiency in cognitive functioning have survival advantage over the other groups with frailty. The four domains are weakly correlated and are independent of each other for mortality prediction. The effects of the four domains seem to be cumulative without interactions. We are worried that the diagnosis of frailty may take distinct populations as equal and assign irrelevant interventions to individuals without corresponding conditions. For example, individuals can have no deficiency in nutritive functioning and be diagnosed with frailty because of the deficiencies in cognitive functioning and sensory problems, but treated with nutritional interventions for frailty. We have recognized several reasons why composite diagnostic criteria can lead to inferior predictive power, poor interpretability of the diagnoses, and inadequate treatment plans. We are concerned that these issues caused by composite diagnostic criteria in frailty can also be found in other medical diagnoses. We proposed a subjective frailty assessment scale that avoids the issues related to composite diagnostic criteria and think it necessary to review the appropriateness and adequacy of the use of composite diagnostic criteria in frailty diagnoses.

## Methods

A variety of frailty indices have been proposed and used in different contexts^11,30^. However, not many of them were compared to each other directly using the same data sources. Three of the most commonly used indices for frailty diagnosis were reviewed using the wave 2004 data of the Health and Retirement Study (HRS)^12,13^. The three frailty indices were continuous measures derived from multiples domains or items^12^. Frailty indices could be categorized into two classes (frailty or not) or three classes (frail or pre-frailty or non-frail in some models) to determine frailty status^12,13^.

The HRS is an ongoing longitudinal survey conducted in the United States^12,28,29^. The first wave of the HRS was conducted in 1996 and repeated about every two years^26–29^. Americans aged 51 years and over were sampled and continuously followed up^26–29^. The spouses of the participants that were younger than the inclusion criteria might be also interviewed and retained in the HRS^26–29^. The details of the design of the HRS could be found elsewhere^13,26–29^. The HRS data with contribution from RAND (version P) that pooled all waves were used for longitudinal analysis^27^. Certain variables that existed only in the 2004 wave were reintroduced from original waves for the diagnosis of frailty^12^. The list of the input variables for the three frailty indices were recently published^12^.

Prior to interview, the HRS participants provided informed consents and were followed by the study team^12,28,29^. The HRS data are publicly accessible and can be obtained via the University of Michigan website^27^. The authors did not have special access to the HRS data and used the version available to the public. The HRS data obtained by the authors were anonymized without any information to identify the participants^27^. This study analyzed the HRS data only without involving human participants and was approved by the ethics committee at the Centre Hospitalier de l’Université de Montréal. This study did not use materials that require authorization or permission from the participants.

### Frailty indices

Three of the most commonly used frailty indices were compared to each other in previous studies: the Functional Domain Model proposed by Strawbridge *et al*. (1998)^31^, the Burden Model by Rockwood *et al*. (2007), and the Biological Syndrome Model by Fried *et al*. (2004)^12,13^. The three frailty indices were the sums of the respective sets of input domains or diagnostic criteria^12,13^. Each domain or criterion could be scored between zero and one^13^. For most domains, the domains with score one represented the presence of the deficiency and score zero suggested the absence^12,13^. For example, the “sensory problems” domain was scored one when individuals met the criteria, having poor eyesight or poor hearing^12,13^. The numbers of domains or diagnostic criteria for respective frailty indices were four, 70, and five^12,13^. The frailty thresholds for the three indices were two, 20% of the number of the input domains, and three respectively^12,13^. The individuals with the frailty indices equal to or higher than the respective thresholds were considered frail.

For each frailty index, the HRS participants could be categorized into subgroups according to the combinations of domains or diagnostic criteria. For example, 16 groups could be generated based on the presence or absence of the deficiencies defined by the four diagnostic domains according to the Functional Domain Model (2^4^)^12,13^. The numbers of the possible combinations of the presence or absence of the diagnostic criteria of the three frailty indices were 16 (2^4^), 2^70^, and 32 (2^5^) respectively.

The Functional Domain Model was chosen for evaluation in this study for several reasons. This frailty model, whose conceptualization was fundamental to other frailty measures, remained relevant in recent studies^31,32^. This model had been used to derive important statistics, such as global burden of disease^33^. The number of population groups for comparison according to the presence or absence of the frailty domains, 16, was smaller than those in the other two frailty models, 32 or 2^70^. The four domains of the Functional Domains Model for the diagnosis of frailty are Domain 1) physical functioning, Domain 2) nutritive functioning, Domain 3) cognitive functioning, and Domain 4) sensory problems^12,13^. The domain numbering was used consistently throughout this study. In the figures, the presence and absence of deficiencies in the four domains were represented with four-digit codes. The first digit to the left represented the first domain and the second represented the second domain. This principle was applied to the third and fourth digits. Zero and one in the four-digit codes denoted the presence and absence of deficiencies defined by the four domains respectively. For example, the code, 0011, represents the absence of deficiencies in Domain 1) physical functioning and Domain 2) nutritive functioning and the presence of deficiencies in Domain 3) cognitive functioning and Domain 4) sensory problems. The population groups and the combinations of the domains they presented according to the Functional Domain Model were listed in Fig. 1. Among the 16 groups, one group did not present any deficiencies in the four frailty domains (group 1: 0000) and another presented four deficiencies in all domains (group 16: 1111). Four, six and four groups presented one, two, and three deficiencies in the four domains respectively.

### Characteristics and survival patterns

The characteristics of the HRS participants were compared across the 16 groups, including age, sex, race, education, per capita income, per capita wealth, and survival pattern. Survival was chosen for several reasons. The HRS is a health-oriented study and health outcomes, including survival, are well recorded and validated in previous studies^12^. However, due to its long history, health outcomes that were emphasized in recent studies might not be readily available in the data set, particularly quality of life^34^. As a result, survival was adopted as the main health outcome for this study. Race was categorized into white/Caucasian, black/African American, and others^26–29^. Educational attainment was reported as years of education^28,29^. The differences in continuous variables between any two groups were examined with t tests^35^. Whether the values of continuous variables were the same across more than two groups were tested with one-way analysis of variance^35^. The differences in proportions between any two groups were analyzed with Chi-square tests^35^. Whether the proportions across more than two groups were the same was tested with Kruskal–Wallis test^36^.

The associations and their 95% confidence intervals (CIs) of the four frailty domains with numerical variables were also quantified using regression models. The four domains were the independent variables and the characteristics were the dependent variables. The relationships between frailty domains and dichotomous variables were analyzed with logistic regression. The odds ratios (ORs) and 95% CIs associated with the frailty domains were derived.

The associations between the four input domains, the frailty index and the frailty status defined by the Functional Domains Model were analyzed with Spearman’s correlation^37,38^. The strength of correlation was categorized as following: 0 to 0.19 as very weak, 0.20 to 0.39 as weak, 0.40 to 0.59 as moderate, 0.60 to 0.79 as strong, and 0.80 to 1 as very strong^39^. The survival patterns of the groups were described with Kaplan-Meier survival functions^12,40^. The differences between survival curves were tested with log-rank tests and p values were derived according to Chi-square and degrees of freedom^40^. The survival curves of the 16 groups, groups with any one deficiency in the four domains, groups with any two, groups with any three, and groups with two or more were also compared with each other via log-rank tests^40^. The mortality risks of the combinations of the diagnostic domains were compared with survival analysis, while controlling for demographic characteristics: age, sex, race, education in years, per capita income and per capita wealth^41^. However, due to the violation of the proportional hazard assumption, Cox proportional survival analysis was not conducted and discrete-time survival analysis was used instead^12,40^. P values less than 0.05 were considered statistically significant, two-tailed. The p values derived from multiple comparisons were adjusted based on the Benjamini-Hochberg method^37,38^. All statistical analyses and data processing were implemented within R environment (v3.31)^42^ and RStudio (v1.0.44)^43^.

### Expected results

When a group of individuals were diagnosed with the same disease, they were likely to be regarded as experiencing similar pathological process and treated in the same way by physicians. Two statuses were defined based on frailty diagnosis: frailty and non-frailty. The characteristics of all population groups, diagnosed with frailty or not, were expected to be different. If the diagnosis of frailty did not fail to classify individuals with different disease trajectories, the characteristics of the 11 population groups diagnosed with frailty were not expected to be significantly different from each other. The characteristics of the five population groups regarded as non-frail were not expected to be significantly different from each other either. The characteristics that were compared between populations groups were age, sex, race, education, per capita income, per capita wealth, and survival patterns.

### Ethics approval

This secondary data analysis was approved by the ethics review committee at the Centre Hospitalier de l’Université de Montréal. All methods were performed in accordance with the guidelines and regulations relevant to the analysis of public data.

### Consent to participate

The consents to participate from the Health and Retirement Study participants that we are unable to identify are not required by the ethics committee for this secondary data analysis project.

## Supplementary information

Supplementary information

Supplementary information2

Supplementary information3

Supplementary information4

## Data Availability

The HRS data produced by the RAND Center for the Study of Aging can be accessed via the University of Michigan site (https://hrs.isr.umich.edu/data-products). The authors do not have special access to the HRS data. The authors do not have access to identifying patient data and are unable to retrieve patients’ identification. The data available to the authors are anonymized and there are no identifiers available.
