# Supplementary material for: Composite diagnostic criteria are problematic for linking potentially distinct populations: the case of frailty
Source: Sci Rep. 2020 Feb 13;10:2601. doi: 10.1038/s41598-020-58782-1 (PMC7018968; doi:10.1038/s41598-020-58782-1)
Supplement: Supplementary file 1 — Supplementary information [file 41598_2020_58782_MOESM1_ESM.docx]

# Supplemental materials

Appendix 1: comparison in the characteristics of the 16 groups of Health and Retirement Study participants categorized based on the presence or absence of deficiency in the four frailty domains of the Functional Domains Model.

Appendix 2: the regression coefficients of the four frailty domains for the estimation of individual characteristics.

Appendix 3: the Odds ratios of frailty index, frailty status, frailty domains, and interactions between frailty domains for the mortality prediction.

­­­­­­­­­­­­
