# Supplementary material for: Composite diagnostic criteria are problematic for linking potentially distinct populations: the case of frailty
Source: Sci Rep. 2020 Feb 13;10:2601. doi: 10.1038/s41598-020-58782-1 (PMC7018968; doi:10.1038/s41598-020-58782-1)
Supplement: Supplementary file 2 — Supplementary information2 [file 41598_2020_58782_MOESM2_ESM.pdf]

Composite diagnostic criteria are problematic for linking potentially distinct populations: the case of frailty

Authors
Yi-Sheng Chao, Chao-Jung Wu, Hsing-Chien Wu, Hui-Ting Hsu, Lien-Cheng Tsao, Yen-Po Cheng, Yi-Chun Lai, Wei-Chih Chen

Supplemental material

Groups	1 (0000)	2 (0001)	3 (0010)	4 (0011)	5 (0100)	6 (0101)	7 (0110)	8 (0111)	9 (1000)	10 (1001)	11 (1010)	12 (1011)	13 (1100)	14 (1101)	15 (1110)	16 (1111)	Total	P for all	P for one-domain groups	P for two-domain groups	P for three-domain groups	P for two or more-domain groups
**n**	4084	1901	135	178	221	129	16	41	1686	1717	139	377	155	189	34	78	11113					
**Agg (years)**	72.8	74.8	79.6	80.1	74	77.1	80.2	85.6	74.3	76.6	80.8	82.3	74.6	77.8	83.2	83.7	74.9	0	0.2	0.1	0	0.1
**(SD)**	'(6.3)	'(7.3)	'(8.3)	'(9.1)	'(7.6)	'(8.7)	'(8.3)	'(7.9)	'(6.9)	'(7.8)	'(7.7)	'(8.6)	'(7.7)	'(8.8)	'(8)	'(8.9)	'(7.6)					
**Female (%)**	0.55	0.42	0.64	0.43	0.71	0.65	0.88	0.76	0.69	0.6	0.68	0.58	0.79	0.72	0.85	0.74	0.57	0	0	0	0	0
**Race: white (%)**	0.87	0.85	0.59	0.71	0.81	0.8	0.62	0.71	0.84	0.82	0.73	0.74	0.85	0.84	0.74	0.77	0.84	0	0	0.01	0.71	0.02
**Education (years)**	12.7	11.5	9.5	8.7	12.4	11.5	10	10.4	12.3	11.3	9.3	8.8	11.7	10.8	11	9.2	11.8	0	0	0	0	0.5
**(SD)**	'(2.9)	'(3.6)	'(4.1)	'(4.5)	'(3.1)	'(3.3)	'(4)	'(3.4)	'(3.1)	'(3.3)	'(4.1)	'(4.4)	'(3.3)	'(3.7)	'(4.5)	'(3.8)	'(3.4)					
**Wealth ($)**	295700.8	230560.7	115436.7	84109.9	229480.2	200009	177983.3	84782.3	244061.6	172882.5	112866.3	97021.5	167961.3	100692.4	41960.1	186132.2	232907.6	0	0.2	0.9	0.6	0.1
**(SD)**	'(604553.2)	'(553481.4)	'(241695.4)	'(191269.1)	'(412951.5)	'(495543.8)	'(304334.1)	'(269469.7)	'(611158.7)	'(522377.3)	'(286774.2)	'(266054.3)	'(432340.8)	'(214880.3)	'(82164.6)	'(1099776.2)	'(559639.3)					
**Income ($)**	29967.3	24314	15523.1	11960.6	26180.2	18419	14081	12637	25033.5	19901.7	12628.6	14277.4	26345.4	17791.7	11621.1	13919.6	24753.8	0	0.8	0.3	0.4	0.5
**(SD)**	'(46461.3)	'(36616.8)	'(22947.6)	'(10379.2)	'(32880.4)	'(15909.1)	'(14714.3)	'(16219.6)	'(41458.7)	'(48623.1)	'(11691.1)	'(18613.8)	'(89634.4)	'(43627.9)	'(10648)	'(27233.6)	'(43279.5)					
**Mean follow-up time (days)**	3063.8	2871.2	2036.4	1734.4	2790.1	2467.1	1919.4	1237.2	2772.6	2494.9	1601.7	1429	2509	1972.4	1280.1	1080	2724.5					
**(SD)**	'(949.3)	'(1064.1)	'(1147.4)	'(1252.6)	'(1109.6)	'(1308)	'(1361.6)	'(1014.1)	'(1115.4)	'(1217.2)	'(1173.2)	'(1163.1)	'(1221.9)	'(1228.2)	'(1241.8)	'(994.1)	'(1162.6)					
**Follow-up time for surivals (days)**	3479.8	3487.2	3482.9	3458.7	3496.1	3503	3606	3157.5	3457.4	3449.8	3451.2	3395.5	3468.5	3416.9	3553.8	3662.7	3472.5	0	0	0	0	0
**(SD)**	'(512.2)	'(521.6)	'(570.6)	'(586.2)	'(498.2)	'(566.3)	'(128.2)	'(340.2)	'(567.8)	'(602)	'(437)	'(642.3)	'(601.7)	'(622.2)	'(311.1)	'(35.2)	'(538.4)					
**Death (%)**	0.3	0.4	0.7	0.8	0.4	0.6	0.8	0.9	0.4	0.6	0.9	0.9	0.6	0.7	0.9	1	0.4					
**Follow-up time for the deceased (days)**	2094.5	1984.3	1544.6	1278.6	1858.8	1635.5	1357.2	1029.6	1902	1762.6	1290.9	1133.4	1797.6	1480.6	977	976.7	1799.2					
**(SD)**	'(1023.3)	'(1019)	'(835)	'(947.1)	'(1001.7)	'(1127.8)	'(1069.8)	'(824)	'(1030.6)	'(1050.8)	'(949.6)	'(908.3)	'(1069.2)	'(967.8)	'(967.7)	'(864.4)	'(1056.7)					
