# Supplementary material for: Composite diagnostic criteria are problematic for linking potentially distinct populations: the case of frailty
Source: Sci Rep. 2020 Feb 13;10:2601. doi: 10.1038/s41598-020-58782-1 (PMC7018968; doi:10.1038/s41598-020-58782-1)
Supplement: Supplementary file 3 — Supplementary information3 [file 41598_2020_58782_MOESM3_ESM.pdf]

Composite diagnostic criteria are problematic for linking potentially distinct populations: the case of frailty

Authors

Yi-Sheng Chao, Chao-Jung Wu, Hsing-Chien Wu, Hui-Ting Hsu, Lien-Cheng Tsao, Yen-Po Cheng, Yi-Chun Lai, Wei-Chih Chen

Supplemental material

Supplemental material 2: associations between frailty domains and demographic characteristics

1) Regression coefficients to predict per capita wealth

	Estimate	Std..Error	t.value	Pr...t..	upper	lower	Coefficients	name	sigtofrail1_3	Domains
(Intercept)	291702.0988	7812.622205	37.33728461	1.69E-287	307014.8383	276389.3593	291702.0988	(Intercept)	NA	(Intercept)
r7frail1_1	-50696.96392	11149.83347	-4.546880817	5.50E-06	-28843.29032	-72550.63753	-50696.96392	r7frail1_1	0.112048428	1.physical functioning
r7frail1_2	-46423.10491	19895.99634	-2.333288775	0.019650799	-7426.952097	-85419.25773	-46423.10491	r7frail1_2	0.24916012	2.nutritive functioning
r7frail1_3	-104958.885	18952.52067	-5.537990793	3.13E-08	-67811.94446	-142105.8255	-104958.885	r7frail1_3	NA	3.cognitive functioning
r7frail1_4	-61716.2145	11068.02636	-5.576081273	2.52E-08	-40022.88284	-83409.54616	-61716.2145	r7frail1_4	0.375036777	4.sensory problems

2) Regression coefficients to predict per capita income

	Estimate	Std..Error	t.value	Pr...t..	upper	lower	Coefficients	name	sigtofrail1_3	Domains
(Intercept)	29516.08975	603.9611194	48.87084417	0	30699.85354	28332.32596	29516.08975	(Intercept)	NA	(Intercept)
r7frail1_1	-3971.866671	861.946953	-4.608017531	4.11E-06	-2282.450643	-5661.282698	-3971.866671	r7frail1_1	0.011702844	1.physical functioning*
r7frail1_2	-2251.661209	1538.076193	-1.463946467	0.143236957	762.968129	-5266.290548	-2251.661209	r7frail1_2	0.006607573	2.nutritive functioning*
r7frail1_3	-9496.616376	1465.140038	-6.481712414	9.45E-11	-6624.9419	-12368.29085	-9496.616376	r7frail1_3	NA	3.cognitive functioning
r7frail1_4	-5219.222512	855.6227874	-6.099910602	1.10E-09	-3542.201849	-6896.243176	-5219.222512	r7frail1_4	0.102924587	4.sensory problems

3) Regression coefficients to predict years of education

	Estimate	Std..Error	t.value	Pr...t..	upper	lower	Coefficients	name	sigtofrail1_3	Domains
(Intercept)	12.64818044	0.046000059	274.9600938	0	12.73834055	12.55802032	12.64818044	(Intercept)	NA	(Intercept)
r7frail1_1	-0.346434747	0.065643954	-5.277481443	1.33E-07	-0.217772598	-0.475096896	-0.346434747	r7frail1_1	5.93E-57	1.physical functioning*
r7frail1_2	-0.129780213	0.117134602	-1.107957944	0.267904043	0.099803608	-0.359364034	-0.129780213	r7frail1_2	1.16E-44	2.nutritive functioning*
r7frail1_3	-2.521831192	0.111579881	-22.60112808	1.31E-110	-2.303134624	-2.74052776	-2.521831192	r7frail1_3	NA	3.cognitive functioning
r7frail1_4	-1.069143429	0.065162502	-16.40734149	8.58E-60	-0.941424924	-1.196861934	-1.069143429	r7frail1_4	1.31E-25	4.sensory problems*

4) Regression coefficients to predict age in years

	Estimate	Std..Error	t.value	Pr...t..	upper	lower	Coefficients	name	sigtofrail1_3	Domains
(Intercept)	72.77006398	0.10034705	725.1838893	0	72.9667442	72.57338377	72.77006398	(Intercept)	NA	(Intercept)

r7frail1_1	1.565645579	0.143210931	10.93244469	1.12E-27	1.846339003	1.284952155	1.565645579	r7frail1_1	4.80E-53	1.physical functioning*
r7frail1_2	1.430079785	0.255548584	5.596117049	2.24E-08	1.930955009	0.929204562	1.430079785	r7frail1_2	1.34E-36	2.nutritive functioning*
r7frail1_3	6.142783448	0.243430373	25.23425228	1.17E-136	6.619906979	5.665659916	6.142783448	r7frail1_3	NA	3.cognitive functioning
r7frail1_4	2.121764623	0.142160182	14.92516817	6.90E-50	2.400398579	1.843130666	2.121764623	r7frail1_4	1.15E-40	4.sensory problems*

5) Log odds ratios to predict being white versus other races or ethnicity

	Estimate	Std..Error	z.value	Pr...z..	upper	lower	Coefficients	name	sigtofrail1_3	Domains
(Intercept)	1.812384703	0.039568941	45.80321461	0	1.889939828	1.734829579	1.812384703	(Intercept)	NA	(Intercept)
r7frail1_1	-0.103908496	0.053993564	-1.924460771	0.054296853	0.00191889	-0.209735883	-0.103908496	r7frail1_1	3.41E-10	1.physical functioning*
r7frail1_2	-0.102398247	0.091916474	-1.114035847	0.265263809	0.077758042	-0.282554536	-0.102398247	r7frail1_2	1.14E-06	2.nutritive functioning*
r7frail1_3	-0.761168765	0.077751986	-9.789701885	1.25E-22	-0.608774872	-0.913562658	-0.761168765	r7frail1_3	NA	3.cognitive functioning
r7frail1_4	-0.105109708	0.053788233	-1.954139463	0.050684726	0.000315228	-0.210534645	-0.105109708	r7frail1_4	5.45E-10	4.sensory problems*

6) Log odds ratios to predict being female

	Estimate	Std..Error	z.value	Pr...z..	upper	lower	Coefficients	name	sigtofrail1_3	Domains
(Intercept)	0.203156973	0.028477763	7.133881145	9.76E-13	0.258973388	0.147340559	0.203156973	(Intercept)	NA	(Intercept)
r7frail1_1	0.6225813	0.04194899	14.84138951	7.91E-50	0.70480132	0.54036128	0.6225813	r7frail1_1	5.28E-10	1.physical functioning*
r7frail1_2	0.733399625	0.080933589	9.061746029	1.28E-19	0.892029459	0.574769791	0.733399625	r7frail1_2	1.22E-08	2.nutritive functioning*
r7frail1_3	0.061598392	0.071055004	0.866911389	0.385990563	0.200866199	-0.077669415	0.061598392	r7frail1_3	NA	3.cognitive functioning
r7frail1_4	-0.477177821	0.041159238	-11.5934561	4.45E-31	-0.396505714	-0.557849928	-0.477177821	r7frail1_4	3.24E-09	4.sensory problems*
