# Supplementary material for: Composite diagnostic criteria are problematic for linking potentially distinct populations: the case of frailty
Source: Sci Rep. 2020 Feb 13;10:2601. doi: 10.1038/s41598-020-58782-1 (PMC7018968; doi:10.1038/s41598-020-58782-1)
Supplement: Supplementary file 4 — Supplementary information4 [file 41598_2020_58782_MOESM4_ESM.pdf]

Composite diagnostic criteria are problematic for linking potentially distinct populations: the case of frailty

Authors

Yi-Sheng Chao, Chao-Jung Wu, Hsing-Chien Wu, Hui-Ting Hsu, Lien-Cheng Tsao, Yen-Po Cheng, Yi-Chun Lai, Wei-Chih Chen

Supplemental material

	Estimate	Std..Error	t.value	Pr...t..	Models	name	deviance	Odds_ratios	upper	lower
factor(year)1	-10.682555	0.1852029	-57.680279	0	Combination	Year 1	33955.7474	2.29E-05	3.30E-05	1.60E-05
factor(year)2	-10.44096	0.18327353	-56.969275	0	Combination	Year 2	33955.7474	2.92E-05	4.18E-05	2.04E-05
factor(year)3	-10.413792	0.18277671	-56.975485	0	Combination	Year 3	33955.7474	3.00E-05	4.29E-05	2.10E-05
factor(year)4	-10.139656	0.18065622	-56.126802	0	Combination	Year 4	33955.7474	3.95E-05	5.63E-05	2.77E-05
factor(year)5	-10.138671	0.18026242	-56.243952	0	Combination	Year 5	33955.7474	3.95E-05	5.63E-05	2.78E-05
factor(year)6	-9.9231812	0.17874208	-55.51676	0	Combination	Year 6	33955.7474	4.90E-05	6.96E-05	3.45E-05
factor(year)7	-9.8292546	0.17785197	-55.266492	0	Combination	Year 7	33955.7474	5.39E-05	7.63E-05	3.80E-05
factor(year)8	-9.7151776	0.17703169	-54.878184	0	Combination	Year 8	33955.7474	6.04E-05	8.54E-05	4.27E-05
factor(year)9	-9.6310267	0.17649365	-54.568687	0	Combination	Year 9	33955.7474	6.57E-05	9.28E-05	4.65E-05
factor(year)1	-9.5377884	0.17632454	-54.092233	0	Combination	Year 10	33955.7474	7.21E-05	0.00010183	5.10E-05
factor(year)1	-10.312328	0.19999992	-51.561663	0	Combination	Year 11	33955.7474	3.32E-05	4.92E-05	2.24E-05
factor(year)1	-18.47139	72.3632849	-0.2552591	0.79852356	Combination	Year 12	33955.7474	9.51E-09	3.76E+53	2.40E-70
factor(group)	0.14548294	0.04819672	3.01852381	0.00254083	Combination	Group 02(00	33955.7474	1.156598	1.27118369	1.05234118
factor(group)	0.93824948	0.11579163	8.10291251	5.43E-16	Combination	Group 03(00	33955.7474	2.55550404	3.20656761	2.03663285
factor(group)	1.13736315	0.10194517	11.156616	6.95E-29	Combination	Group 04(00	33955.7474	3.11853441	3.80827256	2.55371871
factor(group)	0.52157311	0.11121829	4.68963422	2.74E-06	Combination	Group 05(01	33955.7474	1.68467575	2.09501553	1.35470709
factor(group)	0.53866192	0.13071527	4.1208798	3.78E-05	Combination	Group 06(01	33955.7474	1.71371225	2.21413953	1.3263887
factor(group)	1.25175706	0.31583031	3.96338486	7.40E-05	Combination	Group 07(01	33955.7474	3.4964811	6.49338826	1.88274282
factor(group)	1.5403902	0.20038616	7.68710873	1.52E-14	Combination	Group 08(01	33955.7474	4.66641074	6.91122617	3.15072733
factor(group)	0.45145895	0.04857688	9.29370047	1.52E-20	Combination	Group 09(10	33955.7474	1.57060195	1.72749025	1.42796203
factor(group)	0.61516973	0.04597743	13.3798205	8.70E-41	Combination	Group 10(10	33955.7474	1.84997057	2.02442456	1.69055009
factor(group)	1.37323341	0.11018219	12.4632976	1.27E-35	Combination	Group 11(10	33955.7474	3.9480959	4.89978095	3.18125674
factor(group)	1.41536024	0.07497444	18.8779023	2.49E-79	Combination	Group 12(10	33955.7474	4.11796965	4.76982754	3.55519647
factor(group)	0.88710398	0.11672464	7.59997201	2.99E-14	Combination	Group 13(11	33955.7474	2.42808766	3.052266	1.93155173
factor(group)	1.15699283	0.09946023	11.6327185	2.96E-31	Combination	Group 14(11	33955.7474	3.18035501	3.86489639	2.61705799
factor(group)	1.61955044	0.22062885	7.34061027	2.14E-13	Combination	Group 15(11	33955.7474	5.05081914	7.78332006	3.27762109
factor(group)	1.75375061	0.14960844	11.7222704	1.04E-31	Combination	Group 16(11	33955.7474	5.77622648	7.74450188	4.30819088
ragender2.fe	-0.4226322	0.03162292	-13.364744	1.07E-40	Combination	ragender2.fe	33955.7474	0.6553196	0.69722207	0.61593543
raracem2.bla	-0.0050307	0.04546246	-0.1106568	0.91188874	Combination	raracem2.bla	33955.7474	0.9949819	1.08771122	0.91015792
raracem3.oth	-0.3843627	0.09752873	-3.9410204	8.12E-05	Combination	raracem3.oth	33955.7474	0.68088441	0.82431181	0.56241288
r7agey_b	0.09557615	0.00211796	45.1264242	0	Combination	r7agey_b	33955.7474	1.10029261	1.10486965	1.09573453
raedyrs	-0.0039794	0.0046605	-0.8538625	0.39318351	Combination	raedyrs	33955.7474	0.99602848	1.00516846	0.98697161
I(h7atota/h7h	-2.06E-07	4.99E-08	-4.1383878	3.50E-05	Combination	(h7atota/h7h	33955.7474	0.99999979	0.99999989	0.9999997
I(h7itot/h7hh	-1.17E-06	5.94E-07	-1.9646056	0.04946306	Combination	(h7itot/h7hh	33955.7474	0.99999883	1	0.99999767
factor(year)1	-10.667275	0.18410821	-57.940243	0	Frailty domai	Year 1	33962.5538	2.33E-05	3.34E-05	1.62E-05
factor(year)2	-10.424182	0.18212047	-57.237837	0	Frailty domai	Year 2	33962.5538	2.97E-05	4.24E-05	2.08E-05
factor(year)3	-10.396415	0.18158691	-57.25311	0	Frailty domai	Year 3	33962.5538	3.05E-05	4.36E-05	2.14E-05
factor(year)4	-10.121617	0.17943503	-56.408254	0	Frailty domai	Year 4	33962.5538	4.02E-05	5.71E-05	2.83E-05
factor(year)5	-10.121134	0.17906864	-56.520974	0	Frailty domai	Year 5	33962.5538	4.02E-05	5.71E-05	2.83E-05
factor(year)6	-9.9061826	0.17753765	-55.797645	0	Frailty domai	Year 6	33962.5538	4.99E-05	7.06E-05	3.52E-05
factor(year)7	-9.8126465	0.17665516	-55.546901	0	Frailty domai	Year 7	33962.5538	5.48E-05	7.74E-05	3.87E-05
factor(year)8	-9.6993582	0.17585126	-55.156603	0	Frailty domai	Year 8	33962.5538	6.13E-05	8.66E-05	4.34E-05
factor(year)9	-9.6156669	0.17531099	-54.849197	0	Frailty domai	Year 9	33962.5538	6.67E-05	9.40E-05	4.73E-05
factor(year)1	-9.5222736	0.17512801	-54.373218	0	Frailty domai	Year 10	33962.5538	7.32E-05	0.00010318	5.19E-05
factor(year)1	-10.294835	0.19888511	-51.762723	0	Frailty domai	Year 11	33962.5538	3.38E-05	4.99E-05	2.29E-05
factor(year)1	-18.471709	72.2798608	-0.2555582	0.79829262	Frailty domai	Year 12	33962.5538	9.50E-09	3.19E+53	2.83E-70
r7frail1_1	0.44438319	0.03143238	14.1377506	2.49E-45	Frailty domai	Domain 1	33962.5538	1.55952797	1.65862784	1.46634912
r7frail1_2	0.45147114	0.05079129	8.88875109	6.29E-19	Frailty domai	Domain 2	33962.5538	1.57062109	1.73502542	1.42179507
r7frail1_3	0.86163845	0.04732544	18.2066648	6.26E-74	Frailty domai	Domain 3	33962.5538	2.36703578	2.59710227	2.15734993
r7frail1_4	0.15025015	0.0317495	4.73236307	2.22E-06	Frailty domai	Domain 4	33962.5538	1.16212492	1.23674035	1.0920112
ragender2.fe	-0.4232557	0.03155933	-13.411429	5.69E-41	Frailty domai	ragender2.fe	33962.5538	0.65491118	0.69670069	0.61562828
raracem2.bla	-0.0030663	0.04532566	-0.0676507	0.94606385	Frailty domai	raracem2.bla	33962.5538	0.99693838	1.08955787	0.91219216
raracem3.oth	-0.3890548	0.09739972	-3.9944143	6.49E-05	Frailty domai	raracem3.oth	33962.5538	0.67769711	0.82024567	0.55992172
r7agey_b1	0.09555823	0.00211327	45.2181589	0	Frailty domai	r7agey_b	33962.5538	1.10027288	1.10483968	1.09572497
raedyrs1	-0.0045007	0.00464	-0.9699755	0.33206138	Frailty domai	raedyrs	33962.5538	0.99550942	1.00460428	0.9864969
I(h7atota/h7h	-2.07E-07	4.98E-08	-4.1627406	3.15E-05	Frailty domai	(h7atota/h7h	33962.5538	0.99999979	0.99999989	0.99999969
I(h7itot/h7hh	-1.17E-06	5.95E-07	-1.9621006	0.04975394	Frailty domai	(h7itot/h7hh	33962.5538	0.99999883	1	0.99999767
factor(year)1	-10.721324	0.18352104	-58.420138	0	Frailty index	Year 1	34110.3606	2.21E-05	3.16E-05	1.54E-05
factor(year)2	-10.493052	0.18160431	-57.779753	0	Frailty index	Year 2	34110.3606	2.77E-05	3.96E-05	1.94E-05

factor(year)3	-10.473744	0.18111016	-57.830789	0	Frailty index	Year 3	34110.3606	2.83E-05	4.03E-05	1.98E-05
factor(year)4	-10.210766	0.17900039	-57.043264	0	Frailty index	Year 4	34110.3606	3.68E-05	5.22E-05	2.59E-05
factor(year)5	-10.216941	0.17864621	-57.190919	0	Frailty index	Year 5	34110.3606	3.65E-05	5.19E-05	2.57E-05
factor(year)6	-10.004768	0.17711712	-56.486732	0	Frailty index	Year 6	34110.3606	4.52E-05	6.39E-05	3.19E-05
factor(year)7	-9.9145524	0.17625382	-56.25156	0	Frailty index	Year 7	34110.3606	4.94E-05	6.99E-05	3.50E-05
factor(year)8	-9.8034834	0.1754321	-55.881923	0	Frailty index	Year 8	34110.3606	5.53E-05	7.79E-05	3.92E-05
factor(year)9	-9.7207756	0.17489149	-55.581753	0	Frailty index	Year 9	34110.3606	6.00E-05	8.46E-05	4.26E-05
factor(year)1	-9.6277575	0.17467174	-55.119148	0	Frailty index	Year 10	34110.3606	6.59E-05	9.28E-05	4.68E-05
factor(year)1	-10.401652	0.19858922	-52.377725	0	Frailty index	Year 11	34110.3606	3.04E-05	4.48E-05	2.06E-05
factor(year)1	-18.685491	71.9111927	-0.2598412	0.79498688	Frailty index	Year 12	34110.3606	7.67E-09	1.25E+53	4.71E-70
r7frailim1	0.40505726	0.01698291	23.8508737	2.50E-125	Frailty index	Frailty index	34110.3606	1.49938835	1.5501377	1.45030047
ragender2.fe	-0.3886296	0.0308587	-12.593842	2.45E-36	Frailty index	ragender2.fe	34110.3606	0.67798533	0.72025745	0.63819418
raracem2.bla	0.03241693	0.04507987	0.71909994	0.47208126	Frailty index	raracem2.bla	34110.3606	1.03294808	1.12836928	0.94559624
raracem3.oth	-0.3327958	0.09688065	-3.4351109	0.00059259	Frailty index	raracem3.oth	34110.3606	0.71691658	0.86683233	0.59292825
r7agey_b2	0.0966089	0.00209526	46.1083325	0	Frailty index	r7agey_b	34110.3606	1.10142952	1.10596207	1.09691554
raedyrs2	-0.0103928	0.00455124	-2.2835051	0.02240302	Frailty index	raedyrs	34110.3606	0.98966103	0.99852873	0.98087208
I(h7atota/h7	-2.06E-07	5.01E-08	-4.1210661	3.77E-05	Frailty index	(h7atota/h7h	34110.3606	0.99999979	0.99999989	0.9999997
I(h7itot/h7hh	-1.13E-06	5.90E-07	-1.9171682	0.05521982	Frailty index	(h7itot/h7hh	34110.3606	0.99999887	1.00000003	0.99999771
factor(year)1	-10.678167	0.18390764	-58.062662	0	Frailty status	Year 1	34284.8083	2.30E-05	3.30E-05	1.61E-05
factor(year)2	-10.459911	0.18208417	-57.445473	0	Frailty status	Year 2	34284.8083	2.87E-05	4.10E-05	2.01E-05
factor(year)3	-10.447189	0.18164489	-57.514355	0	Frailty status	Year 3	34284.8083	2.90E-05	4.14E-05	2.03E-05
factor(year)4	-10.191149	0.17960043	-56.743455	0	Frailty status	Year 4	34284.8083	3.75E-05	5.33E-05	2.64E-05
factor(year)5	-10.202175	0.17926742	-56.910371	0	Frailty status	Year 5	34284.8083	3.71E-05	5.27E-05	2.61E-05
factor(year)6	-9.9929487	0.17776174	-56.215406	0	Frailty status	Year 6	34284.8083	4.57E-05	6.48E-05	3.23E-05
factor(year)7	-9.9065582	0.17693406	-55.990114	0	Frailty status	Year 7	34284.8083	4.98E-05	7.05E-05	3.52E-05
factor(year)8	-9.7976238	0.17613723	-55.624947	0	Frailty status	Year 8	34284.8083	5.56E-05	7.85E-05	3.94E-05
factor(year)9	-9.7169497	0.17560333	-55.334655	0	Frailty status	Year 9	34284.8083	6.03E-05	8.50E-05	4.27E-05
factor(year)1	-9.6249003	0.17539429	-54.875791	0	Frailty status	Year 10	34284.8083	6.61E-05	9.32E-05	4.68E-05
factor(year)1	-10.399307	0.19926826	-52.187473	0	Frailty status	Year 11	34284.8083	3.05E-05	4.50E-05	2.06E-05
factor(year)1	-18.643042	72.3454223	-0.2576948	0.79664304	Frailty status	Year 12	34284.8083	8.01E-09	3.06E+53	2.10E-70
I(r7frailim1 >	0.65011669	0.03252099	19.9906807	1.05E-88	Frailty status	Frailty status	34284.8083	1.91576438	2.04185321	1.7974618
ragender2.fe	-0.387107	0.03085048	-12.547843	4.39E-36	Frailty status	ragender2.fe	34284.8083	0.67901844	0.72134334	0.63917695
raracem2.bla	0.0425066	0.04509981	0.94250072	0.34593894	Frailty status	raracem2.bla	34284.8083	1.04342294	1.13985633	0.95514796
raracem3.oth	-0.2888744	0.09675166	-2.9857304	0.0028298	Frailty status	raracem3.oth	34284.8083	0.7491063	0.90552434	0.61970752
r7agey_b3	0.10042681	0.00208113	48.2560016	0	Frailty status	r7agey_b	34284.8083	1.10564272	1.11016185	1.10114198
raedyrs3	-0.0171347	0.00454249	-3.7720942	0.00016199	Frailty status	raedyrs	34284.8083	0.98301126	0.99180236	0.97429808
I(h7atota/h7	-2.30E-07	5.11E-08	-4.4976878	6.88E-06	Frailty status	(h7atota/h7h	34284.8083	0.99999977	0.99999987	0.99999967
I(h7itot/h7hh	-1.12E-06	5.97E-07	-1.8788577	0.06026725	Frailty status	(h7itot/h7hh	34284.8083	0.99999888	1.00000005	0.99999771
factor(year)1	-10.67723	0.18495681	-57.728233	0	Frailty domai	Year 1	33958.0994	2.31E-05	3.31E-05	1.61E-05
factor(year)2	-10.435972	0.18302999	-57.017824	0	Frailty domai	Year 2	33958.0994	2.94E-05	4.20E-05	2.05E-05
factor(year)3	-10.409049	0.18252633	-57.027658	0	Frailty domai	Year 3	33958.0994	3.02E-05	4.31E-05	2.11E-05
factor(year)4	-10.134888	0.18040182	-56.179522	0	Frailty domai	Year 4	33958.0994	3.97E-05	5.65E-05	2.79E-05
factor(year)5	-10.134139	0.18002256	-56.293718	0	Frailty domai	Year 5	33958.0994	3.97E-05	5.65E-05	2.79E-05
factor(year)6	-9.9185477	0.17849793	-55.566739	0	Frailty domai	Year 6	33958.0994	4.93E-05	6.99E-05	3.47E-05
factor(year)7	-9.8248497	0.17761754	-55.314637	0	Frailty domai	Year 7	33958.0994	5.41E-05	7.66E-05	3.82E-05
factor(year)8	-9.7108781	0.17680077	-54.92554	0	Frailty domai	Year 8	33958.0994	6.06E-05	8.57E-05	4.29E-05
factor(year)9	-9.6271306	0.17626923	-54.616058	0	Frailty domai	Year 9	33958.0994	6.59E-05	9.31E-05	4.67E-05
factor(year)1	-9.5338064	0.17608949	-54.141826	0	Frailty domai	Year 10	33958.0994	7.24E-05	0.00010219	5.12E-05
factor(year)1	-10.307612	0.1997762	-51.595794	0	Frailty domai	Year 11	33958.0994	3.34E-05	4.94E-05	2.26E-05
factor(year)1	-18.481369	72.3507636	-0.2554412	0.79838292	Frailty domai	Year 12	33958.0994	9.41E-09	3.63E+53	2.44E-70
r7frail1_11	0.45565342	0.04583357	9.94147694	2.83E-23	Frailty domai	Domain 1	33958.0994	1.57720362	1.72544886	1.44169516
r7frail1_21	0.46958001	0.09267046	5.06720305	4.05E-07	Frailty domai	Domain 2	33958.0994	1.59932236	1.91786791	1.33368518
r7frail1_31	0.9977443	0.09108655	10.9538054	6.64E-28	Frailty domai	Domain 3	33958.0994	2.71215711	3.24227074	2.26871745
r7frail1_41	0.14560855	0.04565567	3.1892767	0.0014268	Frailty domai	Domain 4	33958.0994	1.15674329	1.26502724	1.05772825
I(r7frail1_1 *	0.00638135	0.1026495	0.06216637	0.95043049	Frailty domai	(Domain 1 *	33958.0994	1.00640175	1.23068923	0.82298964
I(r7frail1_1 *	-0.1444056	0.09059913	-1.593896	0.11096294	Frailty domai	(Domain 1 *	33958.0994	0.86553662	1.03372492	0.72471277
I(r7frail1_1 *	0.01433691	0.06219721	0.23050725	0.81769817	Frailty domai	(Domain 1 *	33958.0994	1.01444017	1.14596083	0.898014
I(r7frail1_2 *	-0.1485326	0.12289092	-1.2086537	0.22679918	Frailty domai	(Domain 2 *	33958.0994	0.86197194	1.09673067	0.67746407
I(r7frail1_2 *	0.02030881	0.10277567	0.19760328	0.84335593	Frailty domai	(Domain 2 *	33958.0994	1.02051644	1.24825817	0.83432564
I(r7frail1_3 *	-0.0350484	0.09238121	-0.3793893	0.70439972	Frailty domai	(Domain 3 *	33958.0994	0.96555864	1.15721787	0.80564215

ragender2.fe	-0.4231824	0.03161372	-13.386035	8.01E-41	Frailty domai	ragender2.fe	33958.0994	0.65495918	0.69682605	0.61560777
raracem2.bla	-0.0061333	0.04544154	-0.1349714	0.89263483	Frailty domai	raracem2.bla	33958.0994	0.99388546	1.08646804	0.90919224
raracem3.oth	-0.3848394	0.09745013	-3.9490908	7.85E-05	Frailty domai	raracem3.oth	33958.0994	0.68055991	0.82379203	0.56223145
r7agey_b4	0.09552667	0.00211636	45.1372458	0	Frailty domai	r7agey_b	33958.0994	1.10023816	1.1048115	1.09568376
raedyrs4	-0.0040867	0.00465412	-0.8780902	0.37989718	Frailty domai	raedyrs	33958.0994	0.9959216	1.00504804	0.98687804
I(h7atota/h7l	-2.07E-07	4.99E-08	-4.1475823	3.36E-05	Frailty domai	(h7atota/h7h	33958.0994	0.99999979	0.99999989	0.9999997
I(h7itot/h7hh	-1.16E-06	5.94E-07	-1.9561697	0.05044834	Frailty domai	(h7itot/h7hh	33958.0994	0.99999884	1	0.99999767
